# Healthy cortical development through adolescence and early adulthood

**DOI:** 10.1007/s00429-017-1424-0

**Published:** 2017-04-17

**Authors:** Natalie J. Forde, Lisa Ronan, Marcel P. Zwiers, Lizanne J. S. Schweren, Aaron F. Alexander-Bloch, Barbara Franke, Stephen V. Faraone, Jaap Oosterlaan, Dirk J. Heslenfeld, Catharina A. Hartman, Jan K. Buitelaar, Pieter J. Hoekstra

**Affiliations:** 1 Department of Psychiatry, University of Groningen, University Medical Center Groningen, Groningen, The Netherlands; 2 Department of Cognitive Neuroscience, Radboud University Medical Center, Donders Institute for Brain, Cognition and Behaviour, Nijmegen, The Netherlands; 3 Brain Mapping Unit, Department of Psychiatry, University of Cambridge, Cambridge, UK; 4 Developmental Psychiatry, Department of Psychiatry, University of Cambridge, Cambridge, UK; 5 Department of Psychiatry, Yale University School of Medicine, New Haven, CT USA; 6 Departments of Human Genetics and Psychiatry, Radboud University Medical Center, Donders Institute for Brain Cognition and Behaviour, Nijmegen, The Netherlands; 7 Departments of Psychiatry and of Neuroscience and Physiology, SUNY Upstate Medical University, Syracuse, NY USA; 8 Department of Biomedicine, K.G. Jebsen Centre for Research On Neuropsychiatric Disorders, University of Bergen, Bergen, Norway; 9 Clinical Neuropsychology Section, Vrije Universiteit Amsterdam, Amsterdam, The Netherlands; 10 Karakter Child and Adolescent Psychiatry University Centre, Nijmegen, The Netherlands

**Keywords:** Neuro-development, Cortex, Cortical thickness, Local gyrification index, Intrinsic curvature, Surface area

## Abstract

Adolescence is a period of significant brain changes; however, the effects of age and sex on cortical development are yet to be fully characterized. Here, we utilized innovative intrinsic curvature (IC) analysis, along with the traditional cortical measures [cortical thickness (CT), local gyrification index (LGI), and surface area (SA)], to investigate how these indices (1) relate to each other and (2) depend on age and sex in adolescent cortical development. T1-weighted magnetic resonance images from 218 healthy volunteers (age range 8.3–29.2 years, M[SD] = 16.5[3.4]) were collected at two sites and processed with FreeSurfer and Caret software packages. Surface indices were extracted per cortex area (frontal, parietal, occipital, temporal, insula, and cingulate). Correlation analyses between the surface indices were conducted and age curves were modelled using generalized additive mixed-effect models. IC showed region-specific associations with LGI, SA, and CT, as did CT with LGI. SA was positively associated with LGI in all regions and CT in none. CT and LGI, but not SA, were inversely associated with age in all regions. IC was inversely associated with age in all but the occipital region. For all regions, males had larger cortical SA than females. Males also had larger LGI in all regions and larger IC of the frontal area; however, these effects were accounted for by sex differences in SA. There were no age-by-sex interactions. The study of IC adds a semi-independent, sensitive measure of cortical morphology that relates to the underlying cytoarchitecture and may aid understanding of normal brain development and deviations from it.

**Electronic supplementary material:**

The online version of this article (doi:10.1007/s00429-017-1424-0) contains supplementary material, which is available to authorized users.

## Introduction

The structural changes that occur in the brain during development are yet to be fully characterized. The brain changes dynamically throughout the lifespan with different regions, tissue types, and circuits all having distinct developmental profiles (Giedd and Rapoport 2010). Various underlying mechanisms (e.g., myelination and pruning) and regulatory factors (e.g., expression of growth factors) are involved. Multiple magnetic resonance imaging (MRI) studies of brain development have investigated cortical indices; most frequently volume (Gogtay et al. 2004; Lenroot et al. 2007) but also the indices that contribute to volume—surface area (SA) and cortical thickness (CT) (Sowell et al. 2007; Shaw et al. 2008; Ostby et al. 2009; Raznahan et al. 2011; Brown et al. 2012; Amlien et al. 2016)—and local gyrification index (LGI) (Raznahan et al. 2011; Shaw et al. 2012). Despite this little has been described about the relationship of the different indices to each other or the regional effects of age and sex.

**Fig. 1:**
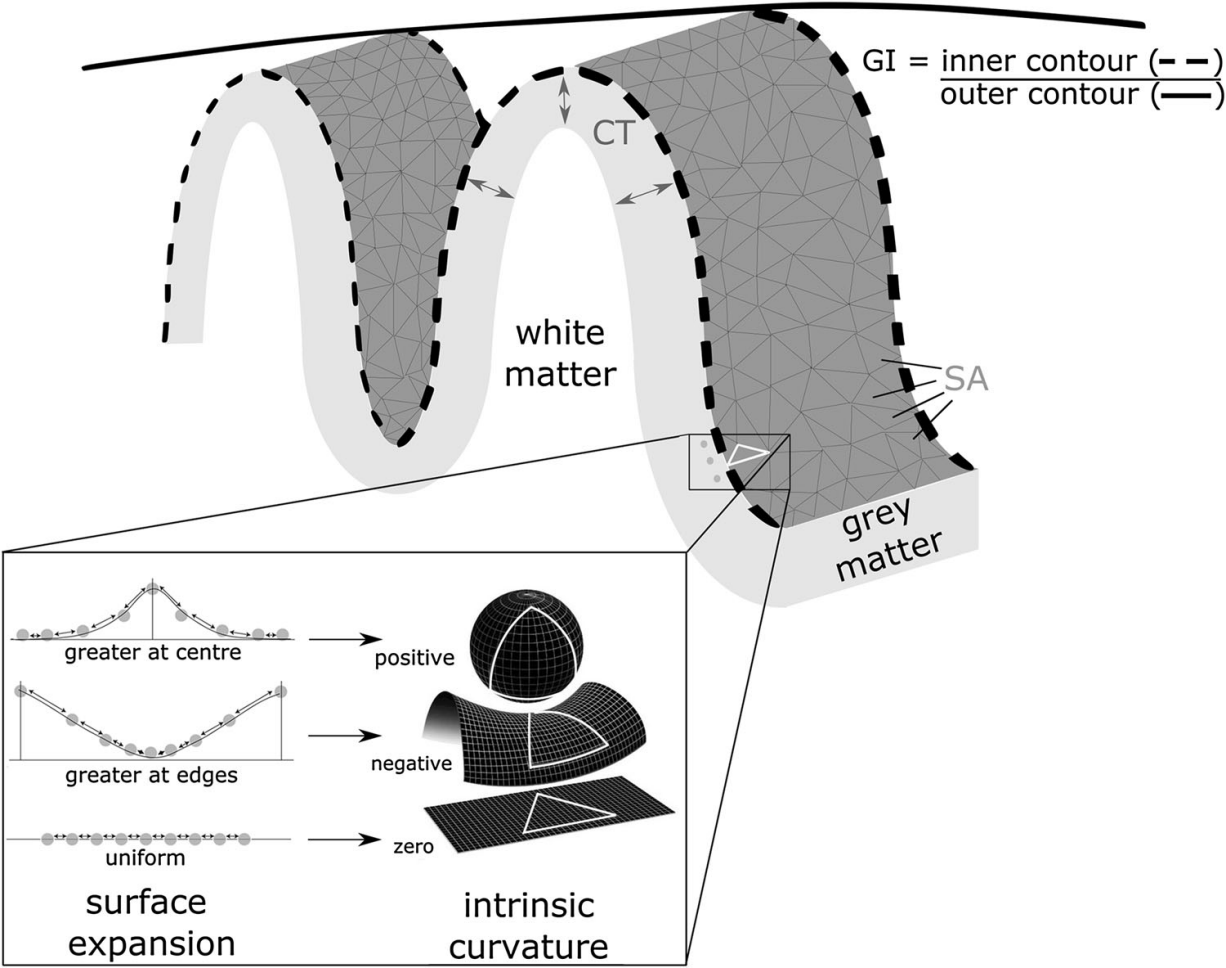
Surface indices. Cortical thickness (CT) is taken as the average shortest distance from the cortical surface to the *grey*/*white* matter border below. Surface area (SA) is the sum of the area of each triangle on the tessellated surface. Gyrification index (GI) is the ratio between the outer hull (*solid line*) and the surface buried within sulci (*broken line*). Intrinsic curvature varies with a higher spatial frequency and reflects a deformation of the surface due to differential expansion. The *grey dots* in the figure above may be thought of as cells within the cortex with the arrows between as connections. Differential expansion of the cortex results in either positive or negative intrinsic curvature values. Uniform expansion generates an overall increase in distances between points but no change in the proportion of long-to-short connections. Differential expansion (positive or negative intrinsic curvature) also increases the overall distance between points, but more importantly, it also increases the relative proportion of short-to-long connections

Here, we aimed to address this shortfall. Furthermore, in addition to the use of traditional cortical indices (CT, SA, and LGI), we also included cortical intrinsic curvature (IC), see Fig. 1. This innovative measure provides information on the intrinsic deformation of the cortical surface and is a function of the surface itself rather than relating to its embedding in 3D space (Pienaar et al. 2008). IC develops due to differential expansion of the cortex during development. It is measured at the millimeter scale and is distinct from, though predictive of, the overall degree of gyrification that is measured at the centimeter scale (Ronan et al. Ronan et al. 2012, 2014). The greater precision of IC increases the power to detect subtle shape differences in the cortex indicative of abnormal neurodevelopment (Ronan et al. 2012). However, the relationship between IC and LGI has not been investigated during development. As mentioned, the mechanism of differential expansion is proposed to be responsible for cortical morphology measured by IC and LGI. The biological determinants of cortical development are complex and highly heritable (Hevner 2006; Ronan and Fletcher 2015; Jernigan et al. 2016). Briefly, various processes including neurogenesis, cell growth, differentiation, apoptosis, and the formation of connections all influence surface expansion with the differing regional expression or rate of these processes resulting in non-uniform or differential expansion of the surface (Ronan and Fletcher 2015). In turn, these processes are governed by a complex array of cellular and molecular (and thus genetic) factors (Hevner 2006; Pontious et al. 2007).

IC can be interpreted in terms of the underlying connectivity of the cortex (Ronan et al. 2011, 2012). Reduced IC has previously been shown to be indicative of decreased short-range cortical connectivity in patients affected by schizophrenia compared with healthy controls (Ronan et al. 2012). Furthermore, a measure related to the intrinsic curvature of the cortical surface, *wiring cost*, was found to be altered in autism spectrum disorders (ASD) (Ecker et al. 2013). Characterizing the developmental trajectory of IC and its relation to other cortical measures in typically developing individuals is an important step in improving our understanding of the mechanisms involved in healthy neurodevelopment. Moreover, this may inform our understanding of abnormal cortical developmental that occurs in disorders like ASD, schizophrenia, and attention-deficit/hyperactivity disorder (ADHD).

We, therefore, used a large cross-sectional MRI data set of typically developing adolescent and young adult volunteers from the NeuroIMAGE project (von Rhein et al. 2014) to determine regional (frontal, parietal, occipital, temporal, cingulate, and insula) effects of age and sex on IC, LGI, CT, and SA, and to determine how these indices relate to each other. We hypothesized that SA would predict both LGI and IC (and IC LGI) in all regions as both LGI and IC develop as a function areal expansion. We did not expect CT and SA to relate given their different genetic underpinnings (Panizzon et al. 2009). For LGI and CT, a general decline as a function of age was expected (Raznahan et al. 2011; Brown et al. 2012; Shaw et al. 2012; Amlien et al. 2016). SA was also expected to differ between males and females in all regions (Raznahan et al. 2011; Amlien et al. 2016). Regarding IC, these analyses were exploratory. Our data set spanned a wide age range from 8 to 29 years; however, the majority of participants were adolescents or young adults.

## Methodology

### Participants

This study was undertaken under the remit of the NeuroIMAGE study; for details, please see von Rhein (von Rhein et al. 2014) and the study Website (www.neuroimage.nl). Briefly, the NeuroIMAGE study is the follow-up, within The Netherlands, of the International Multicenter ADHD Genetics (IMAGE) study (Müller et al. 2011a, b). For the IMAGE study, healthy control families and families who had a child with ADHD combined type were recruited. All participants were Caucasians, aged 6–18 years, and had an IQ ≥70. An extensive battery of diagnostic and neuropsychological tests, as well as genetic data were acquired across the two participating Dutch sites (Vrije Universiteit [VU] in Amsterdam and Radboudumc in Nijmegen). All initial participants were invited to participate in the NeuroIMAGE follow-up study (mean follow up 5.9 years) when neuroimaging data were acquired in addition to a battery of diagnostic and neuropsychological tests similar to that of the initial visit. Exclusion criteria were a diagnosis of autism, epilepsy, and brain or genetic disorders (such as fragile X syndrome). For the current study, individuals with any psychiatric diagnoses, including ADHD, were excluded, as were the siblings of individuals with ADHD. This left 218 participants (126 families) with a mean age of 16.5 years (SD = 3.4, range 8–29) of whom 111 (51%) were male and 107 female. This study was approved by the regional ethics board (CMO Regio Arnhem-Nijmegen) and all participants gave written informed consent. Parents of participants younger than 12 consented for them. Participants between 12 and 18 gave written assent along with their parents written consent.

### Structural MRI acquisition

Two T1-weighted MPRAGE images were acquired for each participant using similar 1.5 Tesla MRI scanners (Siemens SONATA and Siemens AVANTO; Siemens, Erlangen, Germany) and identical head coils (8-channel Phase Array Head Coil). Images were acquired with a sagittal, three-dimensional MPRAGE sequence with the following parameters: TE = 2.95 ms, TR = 2730 ms, TI = 1000 ms, flip angle = 7°, voxel dimension = 1 × 1 × 1 mm, GRAPPA 2, and acquisition time 6.21 min.

### Quality assessment

Image quality was visually inspected for typical imaging artefacts, such as noise, ghosting, and blur, by two independent raters. The better quality scan was selected for each participant and used for further analyses. Those with two poor quality scans were omitted.

### Surface reconstruction

The cortical surfaces were reconstructed using FreeSurfer v5.3 (Fischl et al. 1999b; Dale et al. 1999; Fischl et al. 1999a; Fischl and Dale 2000), a program specifically designed for cortical reconstruction and volumetric segmentation. The fully automated FreeSurfer “recon-all” standard procedure was used (Fischl et al. 1999a, b; Dale et al. 1999; Fischl and Dale 2000) to generate reconstructions of the pial and white matter surfaces for each hemisphere. FreeSurfer surface reconstructions were visually checked to ensure data quality.

### Intrinsic curvature (IC)

IC, which is defined mathematically as the product of the two principle curvatures (these are the maximum and minimum curvatures which occur orthogonally to each other), was calculated per vertex of each participant’s FreeSurfer reconstruction using the Caret software (v5.65, http://brainvis.wustl.edu/wiki/index.php/Caret:About). This process has been detailed previously (Ronan et al. 2014; Forde et al. 2014). The Caret-generated files of IC were imported in MatLab, where they were filtered to remove curvature values that were not consistent with the resolution of cortical reconstruction (Ronan et al. 2012, 2014). Absolute values of the remaining, per-vertex IC measures were calculated. Per region (see Sect. 2.8), we then calculated the skewness of the curvature distribution (Ronan et al. 2012, 2014). Cortical IC has a distribution highly skewed towards zero IC (Pienaar et al. 2008; Ronan et al. 2011, 2012), thus making measures of mean and median less informative. We, therefore, used the dimensionless measure of skew for analysis and inferred that the less skewed the distribution the greater the degree of IC and differential expansion.

### Local gyrification index (LGI)

Gyrification index (GI) is a ratio of the amount of cortical surface exposed as opposed to bury within sulcal folds. A large GI indicates a highly folded surface. LGI quantifies GI at each vertex on the cortical (pial) surface. It was computed in a 3D fashion using a region of interest with diameter of 10 mm around every 100th vertex on the outer surface (outer contour in Fig. 1) before propagating LGI values to each vertex on the pial surface based on their involvement in prior computations and distance to the surface normal of the point on the outer surface where LGI was calculated with FreeSurfer (Schaer et al. 2008).

### Cortical thickness (CT) and surface area (SA)

CT was calculated as the closest distance from the grey/white boundary to the grey/CSF boundary at each vertex on the tessellated surface (Fischl and Dale 2000). SA per region (see “Statistical analysis”) is the sum of the tessellated surface area of vertices contained within that region in mm^2^.

### Statistical analysis

**Fig. 2:**
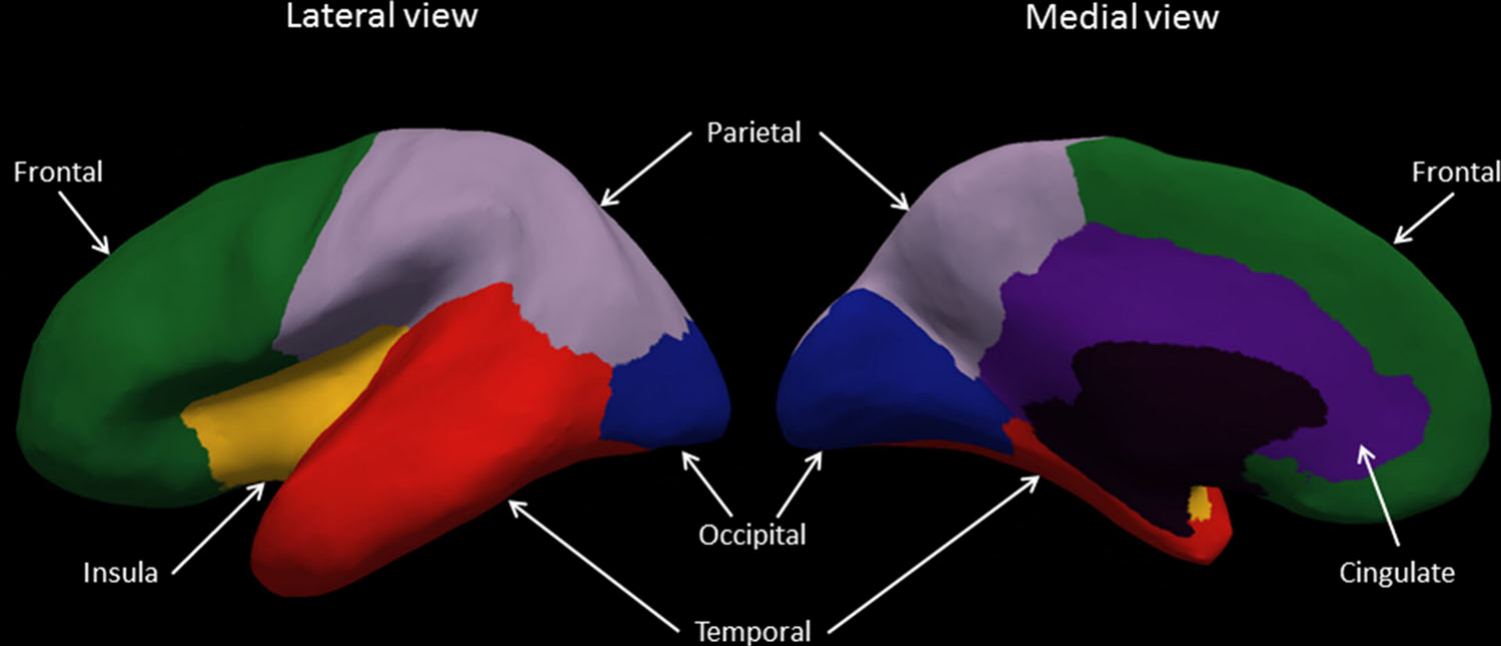
Regions for analysis. Inflated view of the left hemisphere divided into the regions used for analysis. *Left* shows the lateral view and *right* the medial view

To increase the signal-to-noise ratio and reduce the number of statistical evaluations, surface indices were extracted from frontal, parietal, occipital, temporal, cingulate, and insula regions of interest (Fig. 2). These regions were generated by combining several labels from the Desikan–Killiany Atlas (Desikan et al. 2006), which is supplied with the FreeSurfer package. Extraction was performed with the FreeSufer ‘mri_segstats’ function for mean LGI, mean CT, and SA, while IC skew was extracted with MatLab. Indices were averaged across left and right. Where indices differed between hemispheres, they were also subsequently investigated separately. R statistics program (R Core Team 2013) was used for all subsequent statistical analyses. To investigate the relationship between the different surface indices in each region, we used a linear model accounting for both age and sex. We used Bonferroni correction for multiple testing, adjusting our cutoff for statistical significance for these associations (36 tests) to *p* < 0.001. Partial correlations were also undertaken to give an estimate of the strength of the relationship between indices given age and sex separately. IQ was also later included to investigate its influence on the relationship between metrics.

Subsequently, the cortical surface indices were analyzed per region using generalized additive mixed-effect models (GAM; Wood 2006) with the gamm4 (Wood and Scheipl 2014) and mgcv (Wood 2011) packages in R. This allowed the modelling of the relationship between age and our cortical indices with greater flexibility than the standard polynomial form of the growth curve (i.e., age was not restricted to being linear, quadratic, etc.), a method that has previously been effectively applied to neuroimaging data (Alexander-Bloch et al. 2014). Briefly, penalized spline mixed-effect models were used to fit the developmental effects, using age as a smooth factor, for each of our surface indices in each region. Each model also included sex and scanner, and accounted for the non-independence of some subjects (75 sets of siblings) by modelling family as a random factor. Furthermore, IQ [estimated from either WAIS-III (Wechsler 2000) or WISC-III (Wechsler 2002)] and the interaction of age and sex were investigated. Models of CT, LGI, and IC included total SA as an additional covariate to investigate the effect of brain size on findings. Furthermore, intracranial volume was used instead of total SA and yielded similar results (data not shown). We used Bonferroni correction for multiple comparisons, adjusting our cutoff for statistical significance (6 regions × 4 indices = 24 tests) to *p* < 0.002.

## Results

### Demographics

Of the full sample (*n* = 218), 139 data sets were collected in Amsterdam and 79 in Nijmegen. Age distribution was similar across the sites (16.4 years [SD = 3.5] and 16.7 years [SD = 3.2] for Amsterdam and Nijmegen, respectively; Kruskal–Wallis *χ* ^2^ = 1.2, *p* = 0.3). However, on average, female participants were older (17.2 years [SD = 3.4]) compared to male participants (15.8 years [SD = 3.2]; Kruskal–Wallis *χ* ^2^ = 13.2, *p* = 0.0003). Proportionately, more females took part in Nijmegen than Amsterdam (male/female ratio: 32/47 and 79/60, respectively; *χ* ^2^ = 4.7, *p* = 0.03). Average IQ across all participants was 106 (SD = 14), with different mean IQs for the two sites (104 [SD = 13] and 109 [SD = 14] for Amsterdam and Nijmegen, respectively; *χ* ^2^ = 6.9, *p* = 0.01). Males and females did not differ in IQ (*χ* ^2^ = 2.1, *p* = 0.15). The majority of participants were right-handed (right/left/ambidextrous: 190/22/4; *n* = 2 missing data), with no difference between sites (*χ* ^2^ = 4.1, *p* = 0.1) or sexes (*χ* ^2^ = 4.6, *p* = 0.1).

### Associations between the surface indices

Correlations between indices, accounting for age, are shown in Table 1. (Direct and partial correlations for age and sex, separately, can be found in supplementary Table 2) IC in the cingulate and temporal regions differed between the left and right hemispheres, we, therefore, investigated left and right IC separately, as well as their average (Table 1). After accounting for age and sex, the linear model showed that IC and LGI were positively associated in the temporal region (stronger in right hemisphere). There were no significant associations between these measures in the other regions, although analysis of the right cingulate reached significance (*p* < 1 × 10^−3^). IC was positively related to SA in the temporal region. However, an inverse relationship was seen between IC and SA in the cingulate region (stronger in the right temporal region) and no significant associations were seen in the frontal, parietal, occipital, or insula regions. IC was also positively related to CT in frontal, parietal, and cingulate regions (only significant in right cingulate region), while they did not relate significantly in the temporal, occipital, or insular cortex.

**Table 1:** Associations between indices

Index		Frontal	Parietal	Temporal	Occipital	Cingulate	Insula
IC							
LGI		0.16	0.15	0.32**	0.06	−0.23	0.02
SA		0.13	−0.01	0.29*	0.03	−0.33***	−0.07
CT		0.24*	0.24**	0.03	0.05	0.25*	0.03
LGI							
SA		0.59***	0.68***	0.67***	0.65***	0.49***	0.49***
CT		−0.18	−0.12	−0.18	−0.07	−0.16	−0.04
SA							
CT		−0.08	−0.11	−0.14	0.01	−0.31**	−0.02

Estimates from Pearson’s partial correlations between indices accounting for age are shown. Associations accompanied by asterisks reached statistical significance derived from a linear model accounting for both age and sex. Partial correlations accounting for age and sex, separately, can be found in supplementary Table 2. Adjusted significance level is *p* < 0.001. *p < 1 × 10^−3^, **p < 3 × 10^−4^, ***p < 3 × 10^−5^

LGI and SA were strongly positively related in all regions. LGI and CT were not related in any of the regions. There was a negative relationship between CT and SA in the cingulate region, while there were no associations in any of the other regions. In addition, accounting for IQ gave similar results.

### Age and sex effects on the surface indices

**Fig. 3:**
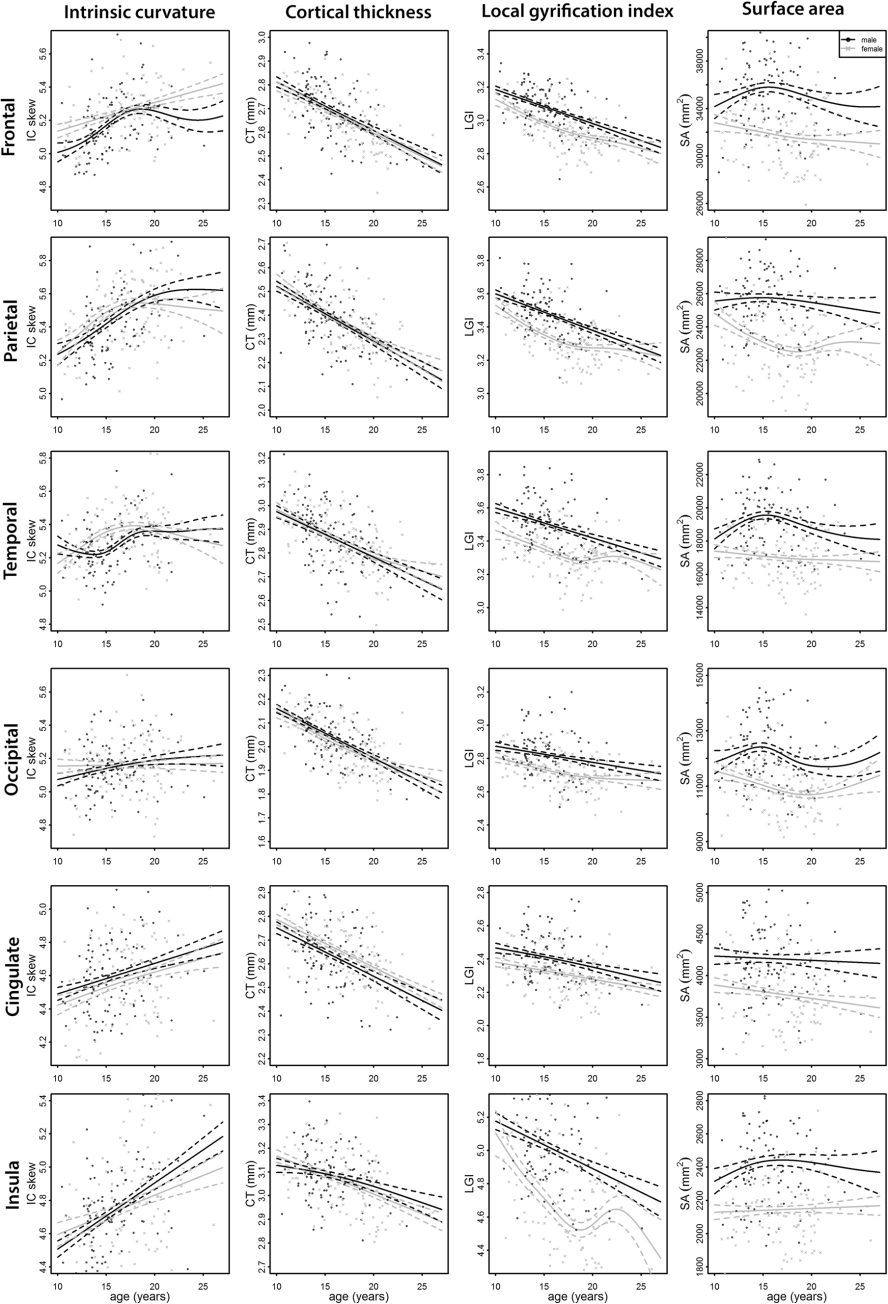
Age curves for each metric per region. *Each graph* plots a metric (*columns from left to right* correspond to intrinsic curvature skew, cortical thickness, local gyrification index, and surface area, respectively) over age separately for males and females in each region (*rows from top to bottom* correspond to frontal, parietal, temporal, occipital, cingulate, and insula regions, respectively). An increase in IC skew indicates a reduction in the degree of IC as a function of age. Males are depicted in *black* with females in* grey*. *Broken lines* represent the standard error for each

Age and sex dependence for each index is presented in Table 2, with the accompanying age curves in Fig. 3. Age was significantly inversely associated with IC in the frontal, parietal, temporal, cingulate, and insula regions but not in the occipital region. In Fig. 3, IC skew is seen to increase with age signifying a reduction in the degree of IC over age. Age was also significantly inversely associated with CT and LGI in all regions but not with SA in any region.

**Table 2:** Association of age and sex with cortical indices

Region	Age F	Age *p*	Sex T	Sex *p*	*R* ^2^ (%)
Intrinsic curvature					
Frontal	38.42	**1.03 × 10** ^**−7**^ ***	−3.42	**7.54 × 10** ^**−4**^ *	24
Parietal	38.23	**7.67 × 10** ^**−8**^ ***	−0.50	0.62	16
Temporal	19.77	**1.89 × 10** ^**−4**^ **	−2.14	0.03	15
Occipital	2.32	0.13	−0.34	0.73	0
Cingulate	19.88	**1.31 × 10** ^**−5**^ ***	2.14	0.03	7
Insula	28.84	**1.95 × 10** ^**−7**^ ***	0.61	0.54	13
Cortical thickness					
Frontal	92.32	**3.77 × 10** ^**−19**^ ***	0.96	0.34	36
Parietal	120.06	**5.11 × 10** ^**−24**^ ***	−0.03	0.97	38
Temporal	61.19	**1.43 × 10** ^**−13**^ ***	−0.42	0.67	25
Occipital	113.54	**7.03 × 10** ^**−23**^ ***	1.72	0.09	37
Cingulate	73.52	**8.39 × 10** ^**−16**^ ***	−2.44	0.02	25
Insula	27.31	**3.94 × 10** ^**−7**^ ***	0.92	0.36	12
Local gyrification index					
Frontal	81.39	**3.29 × 10** ^**−17**^ ***	6.08	**5.57 × 10** ^**−9**^ ***	40
Parietal	84.05	**1.99 × 10** ^**−15**^ ***	7.32	**5.17 × 10** ^**−12**^ ***	42
Temporal	32.94	**3.01 × 10** ^**−8**^ ***	7.85	**1.99 × 10** ^**−13**^ ***	35
Occipital	17.85	**3.50 × 10** ^**−5**^ ***	6.19	**3.04 × 10** ^**−9**^ ***	22
Cingulate	19.50	**1.57 × 10** ^**−5**^ ***	4.85	**2.42 × 10** ^**−6**^ ***	18
Insula	32.05	**4.51 × 10** ^**−8**^ ***	8.10	**4.12 × 10** ^**−14**^ ***	36
Surface area					
Frontal	0.59	0.44	9.18	**3.70 × 10** ^**−17**^ ***	28
Parietal	3.07	0.08	9.87	**3.54 × 10** ^**−19**^ ***	33
Temporal	5.97	0.14	10.32	**1.72 × 10** ^**−20**^ ***	34
Occipital	10.06	0.02	7.94	**1.15 × 10** ^**−13**^ ***	30
Cingulate	0.78	0.38	7.07	**2.13 × 10** ^**−11**^ ***	19
Insula	1.00	0.32	8.61	**1.56 × 10** ^**−15**^ ***	25

Results in bold were statistically significant

Statistics reported are from models including sex, scanner, and familiarity for each index. R^2^ is for the full model. Statistics for LGI, IC, and CT models that also included SA as a covariate were negligibly different regarding age in each case. However, within the LGI model, the inclusion of SA was seen to eliminate all significant effects of sex. Similarly, in the IC model, the significant sex effect in the frontal region disappeared upon inclusion of SA. Adjusted significance level is *p* < 0.002. *p < 2 × 10^−3^, **p < 4 × 10^−4^, ***p < 4 × 10^−5^.

Sex was significantly associated with SA in all regions with males having a larger SA in each region. Males also had significantly larger LGI in all regions and larger IC in the frontal region compared with females. However, when total SA was included in the model, variations in brain size were found to account entirely for these differences (LGI *p* > 0.006, frontal IC *p* = 0.03). There was no significant effect of sex on CT.

There were no age-by-sex interactions for any of the indices in any region (*p*-values > 0.16). Finally, there were no significant influences of IQ (*p* > 0.03) or scanner site (*p* > 0.008) in any region for any of the indices.

## Discussion

We used analysis of IC of the cortical surface, alongside the analysis of more traditional measures of CT, SA, and LGI, to investigate structural brain development during adolescence and early adulthood in a cross-sectional sample of typically developing individuals. This is the first time that IC, CT, LGI, and SA were investigated together in a single study. Studying these indices side by side, with each index derived from a single MR image per subject, allowed for their direct comparison without the confounds of cross-cohort or cross-study comparisons. In addition, it is the first time that IC was investigated in adolescence. Using this multi-index approach, we were able to determine that the indices carried complementary information and related to each other in semi-independent and region-specific manner. Furthermore, each index displays an individual pattern of associations with age and sex.

The current study, partly in line with the previous studies and partly presenting previously undescribed associations, reports a complex pattern of associations between indices. Both LGI and IC were previously shown to correlate positively with SA globally (Ronan et al. 2012), which is expected as both are a function of an increase in SA. This is consistent with the current LGI findings of a strong positive correlation with SA in each region and a positive relationship between IC and SA in the temporal region. However, the findings from the other regions investigated suggest a much more complex relationship between IC and SA than for LGI and SA, with an inverse association in the cingulate and no significant association between IC and SA in the frontal, parietal, occipital, or insula regions. Considering the strong positive relationship between SA and LGI, it is unsurprising that IC relates similarly to both. Here, we saw that IC and LGI are also positively associated in the temporal region and negatively in the cingulate. This is in accordance with the study of Ronan et al. (2014) who showed IC predicted gyrification globally and regionally, with IC and LGI relating positively in most regions (frontal, parietal, temporal, occipital, and insula) but negatively in the cingulate. Similar to the analyses presented here, the former study used a linear model accounting for age and sex. Frontal, parietal, occipital, and insula regions in our study also all showed a positive pattern of association; however, none of these associations were significant. When excluding age from our analyses, we also saw a significant positive association in the frontal and parietal regions (supplementary Table 2). Suggesting that in these regions, the measures change similarly through adolescence, and are potentially driven (or partially driven) by related developmental mechanisms, but that at the average age (16.5 years), there was no significant association between metrics. The insula and occipital regions remained slightly positively associated but non-significant. Intriguingly, these findings suggest that the relationship between IC and SA/LGI is more complex than simply a direct mathematical outcome of the surface expansion. It is postulated that differential expansion of the cortex (indexed by IC) determines the cortical folding pattern (Ronan and Fletcher 2015); however, folding itself may cause compression of certain parts of the surface which, in turn, influences differential expansion (IC). This means that there is not a simple relationship between expansion (SA) and deformation (IC), as illustrated in our results. Furthermore, IC and CT were positively related in the frontal, parietal, and cingulate regions, while there was no significant association between them in the other regions. This has not previously been investigated or reported and again demonstrates the region-specific relationship of IC to the other indices investigated.

CT and SA have previously been shown to be genetically unrelated (Panizzon et al. 2009) and we would not expect them to be related, as is the case for most regions. The consistent pattern of slight negative but non-significant associations may relate to geometric considerations (i.e., space restrictions). The cingulate, which shows a significant negative association, is, perhaps, more likely to display the results of these geometric restrains due to its lesser degree of gyrification. Finally and similarly, CT and LGI were non-significantly but consistently negatively associated in all regions. The complex pattern of region-specific associations and independencies between indices presented here stresses their individual value in research and highlights that each of these indices may be useful in revealing different aspects of the genetic and environmental factors influencing cortical development.

By investigating age effects, our study also revealed that IC, CT, and LGI are all age-dependent, while SA appears stable with age. IC profiles were variable across regions and between the sexes, although in general, IC decreased as a function of age (Fig. 3 shows IC skew increasing, which implies that the degree of IC is decreasing [see methods]); the implications of this are discussed below. CT and LGI also decreased as a function of age in all regions over the age range studied here, 8–29 years. This is in keeping with the previous literature that reported the same, both globally (Shaw et al. 2008; Raznahan et al. 2011; Brown et al. 2012; Shaw et al. 2012) and regionally (CT only: Ostby et al. 2009; Amlien et al. 2016). The previous reports are inconsistent in relation to the development of SA with age. Some have reported that global SA peaks in childhood (8.1 years for females and 9.7 years in males) before decreasing throughout adolescence (Ostby et al. 2009; Shaw et al. 2012), while others have seen little to no decline in SA following the peak (Brown et al. 2012; Amlien et al. 2016) in accordance with the results presented here.

We have shown that IC becomes lower as a function of age in adolescence. IC occurs by way of differential expansion of the cortex during early development. The previous hypotheses and experimental evidence suggested that differential expansion of the cortex, which IC is a quantifiable measure of, relates to both short-range connectivity and the cell density within the cortex; from which it can be inferred that increased IC relates to a higher proportion of short-to-long connections and a lower cell density within the cortex (Ronan et al. 2011, 2012). However, during adolescence, the cortex is no longer expanding, it is, in fact, shrinking in a multi-faceted manner with CT and LGI reducing, as shown in this study and previously (Ostby et al. 2009; Raznahan et al. 2011; Brown et al. 2012; Shaw et al. 2012; Amlien et al. 2016). The changes shown here in IC over adolescence, therefore, are likely related to the way in which the cortex deforms and reduces in size through adolescence rather than cortical expansion. In essence, the lesser the degree of IC, the closer the cortical surface comes to being intrinsically flat. Adolescence is a major period of synaptic pruning and neural reorganization (for review, see Crews et al. 2007). Synaptogenesis in the brain during the early development results in the over-production of synapses to reach levels more than twofold that of adult synapse numbers (Huttenlocher 1979; Petanjek et al. 2011). Following a prolonged period of synaptic pruning that occurs from late childhood through adolescence, these numbers stabilize to adult levels (Huttenlocher 1979; Petanjek et al. 2011). The age at which adult levels are obtained is region-specific; higher order areas like the prefrontal cortex have a very protracted duration of pruning, which only reaches a stable level at around 30 years of age, while most other areas appear to reach maturity earlier (Petanjek et al. 2011). Changes in IC may plausibly be related to these changes in the cytoarchitecture of the cortex during this period.

For sex, we found a large effect on SA, with males having a larger surface area in each region, which is consistent with the previous global findings (Raznahan et al. 2011; Amlien et al. 2016). A similar pattern was seen with LGI, again consistent with the previous literature (Raznahan et al. 2011). However, this effect on LGI could be ascribed to variation in brain size, as the effect was not robust to adding total SA as a covariate to the statistical model. It is important to note, therefore, that studies of LGI should always take the potentially confounding effect of brain size into account. An effect of sex was also present in the frontal IC analysis but again only before accounting for brain size. No effect of sex was found in the analysis of CT, which is consistent with the previous studies to investigate the effect of sex on CT (Raznahan et al. 2011; Amlien et al. 2016). Although more localized, CT differences between the sexes have previously been reported (Sowell et al. 2007).

The different age- and sex-dependent effects on SA and CT that were shown in the current study are in keeping with the genetic study of Panizzon et al. (2009), who found that SA and CT, although both highly heritable, are governed by distinct genetic influences. These genetic findings fit the evidence from this study and others (Ostby et al. 2009; Raznahan et al. 2011; Brown et al. 2012; Amlien et al. 2016), that SA and CT developmental changes are significantly different and strengthen the argument against using the composite measures of volume (a product of SA and CT) as an endophenotype. From the current study, it is difficult to determine regional specificity of trajectories, as we miss or are underpowered at the period of development in which these measures reach their peak (Walhovd et al. 2016). However, region-specific trajectories are likely, given the previous literature on brain volume and cortical thickness indicating maturation to occur earlier in sensory and motor areas compared to more frontal regions (Giedd et al. 1999; Gogtay et al. 2004; Shaw et al. 2008).

As mentioned this is the first study to investigate CT, SA, and LGI concurrently and additionally, the first to examine IC in development. Some limitations must be considered. Low numbers at the extremes of the age range were included resulting in reduced power of our analysis to detect cortical changes in late childhood and early adulthood. Analysis of a restricted age range (10–22 years) indicated that this reduced power at the extremes of the age range did not affect our findings (see supplementary Table 1; supplementary Fig. 1). By not including children younger than eight, this study did not capture the changes that occur before this age. To address the potential confounds of scan site or the age difference between sexes, a matched analysis was also undertaken revealing similar results to the full sample (supplementary Table 3). Furthermore, this was a cross-sectional study and longitudinal studies should be done to confirm and extend our findings regarding age-related associations.

In conclusion, this study adds an innovative cortical measure of IC to the study of cortical development. IC decreases with age during adolescence, which may reflect a decrease in short-range cortico-cortical connectivity and/or a higher cell density. Our findings present a complex pattern of cortical development in which different indices as CT, cortical SA, LGI, and IC have partly different associations with age and sex and relate to each other in a region-specific manner. These findings may form a basis to better understand abnormal cortical development in neurodevelopmental disorders.

## Electronic supplementary material

Below is the link to the electronic supplementary material.

Supplementary material 1 (DOCX 697 kb)
