# Supplementary material for: Healthy cortical development through adolescence and early adulthood
Source: Brain Struct Funct. 2017 Apr 17;222(8):3653–63. doi: 10.1007/s00429-017-1424-0 (PMC5676813; doi:10.1007/s00429-017-1424-0)
Supplement: Supplementary file 1 — Supplementary material 1 (DOCX 697 kb) [file 429_2017_1424_MOESM1_ESM.docx]

**Supplementary Information**

*Age restrictions*

We had few participants at the extremes of our age range (8-29 years). To ensure this was not affecting our findings we also conducted the same analysis on participants aged 10 to 22 years (n=208). This resulted in negligible differences compared to analysis of the full sample (n=218). Findings from the restricted analysis are presented below in supplementary table 1 and supplementary figure 1.

**Supplementary Table 1** Association of age and sex with cortical indices; restricted age range

| Region | Age F | Age *p* | Sex T | Sex *p* | R^2^ (%) |
| --- | --- | --- | --- | --- | --- |
| Intrinsic Curvature | | | | | |
| Frontal | 34.21 | **6.20 x 10^-7^***** | -3.06 | 2.50 x 10^-3^ | 25 |
| Parietal | 32.49 | **3.39 x 10^-7^***** | -0.70 | 0.49 | 18 |
| Temporal | 22.91 | **6.95 x 10^-5^**** | -2.39 | 0.02 | 17 |
| Occipital | 2.97 | 0.09 | -0.23 | 0.82 | 1 |
| Cingulate | 14.70 | **1.67 x 10^-4^**** | 2.28 | 0.02 | 6 |
| Insula | 19.22 | **1.83 x 10^-5^***** | 0.44 | 0.66 | 12 |
| Cortical Thickness | | | | | |
| Frontal | 72.34 | **1.60 x 10^-15^***** | 1.07 | 0.29 | 32 |
| Parietal | 104.90 | **2.93 x 10^-21^***** | 0.24 | 0.81 | 35 |
| Temporal | 49.97 | **1.85 x 10^-11^***** | -0.30 | 0.76 | 23 |
| Occipital | 99.81 | **2.27 x 10^-20^***** | 2.00 | 0.05 | 35 |
| Cingulate | 46.59 | **7.85 x 10^-11^***** | -2.27 | 0.02 | 19 |
| Insula | 15.12 | **1.35 x 10^-4^**** | 0.92 | 0.36 | 7 |
| Local Gyrification Index | | | | | |
| Frontal | 71.42 | **2.34 x 10^-15^***** | 5.82 | **2.20 x 10^-8^***** | 38 |
| Parietal | 79.08 | **1.27 x 10^-14^***** | 7.22 | **1.04 x 10^-11^***** | 42 |
| Temporal | 29.61 | **1.43 x 10^-7^***** | 7.77 | **3.70 x 10^-13^***** | 36 |
| Occipital | 20.25 | **1.12 x 10^-5^***** | 5.97 | **1.03 x 10^-8^***** | 24 |
| Cingulate | 16.20 | **7.94 x 10^-5^**** | 4.73 | **4.20 x 10^-6^***** | 17 |
| Insula | 31.03 | **6.14 x 10^-7^***** | 8.19 | **2.84 x 10^-14^***** | 38 |
| Surface Area | | | | | |
| Frontal | 0.47 | 0.49 | 8.92 | **2.69 x 10^-16^***** | 28 |
| Parietal | 4.52 | 0.03 | 9.55 | **4.23 x 10^-18^***** | 33 |
| Temporal | 5.62 | 0.15 | 9.98 | **2.52 x 10^-19^***** | 35 |
| Occipital | 11.36 | 0.01 | 7.60 | **1.07 x 10^-12^***** | 31 |
| Cingulate | 1.46 | 0.23 | 6.74 | **1.62 x 10^-10^***** | 19 |
| Insula | 1.21 | 0.27 | 8.50 | **4.05 x 10^-15^***** | 26 |

Statistics reported are from models including sex, scanner and familiality for each index for participants within the age range 10-22 years (n=208). R^2^ is for the full model. Statistics for LGI, IC and CT models that also included SA as a covaraiate were negligibly different regarding age. However, within the LGI model the inclusion of SA was seen to eliminate all significant effects of sex. Reults are comparable to that of the full model (n=218, age range = 8-29). Adjusted significance level is *p*<0.002. **p*< 2 x 10^-3^, ***p*< 4 x 10^-4^, ****p*< 4 x 10^-5^.


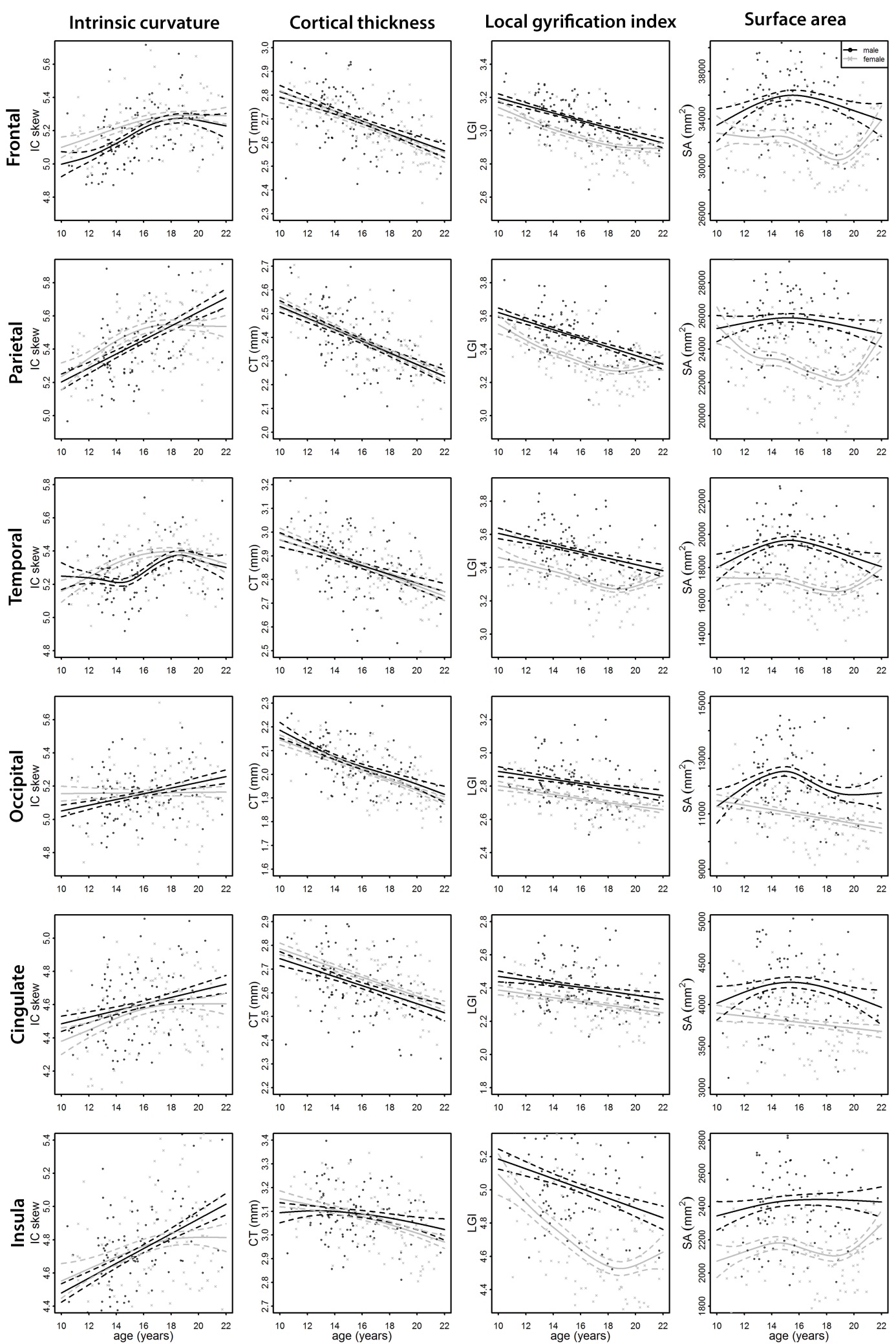


**Supplementary Fig 1** Age-curves for each metric per region; restricted age range

Each graph plots a metric (columns from left to right correspond to intrinsic curvature skew, cortical thickness, local gyrification index and surface area, respectively) over age separately for males and females in each region (rows from top to bottom correspond to frontal, parietal, temporal, occipital, cingulate and insula regions, respectively) . An increase in IC skew indicates a reduction in the degree of IC as a function of age. Males are depicted in black with females in grey. Broken lines represent the standard error for each.

*Partial Correlations*

**Supplementary Table 2** Correlations and partial correlations between indices

| Index | | Frontal | Parietal | Temporal | Occipital | Cingulate | Insula |
| --- | --- | --- | --- | --- | --- | --- | --- |
|  |  | direct correlation | | | | | |
| IC | LGI | 0.34*** | 0.31*** | 0.40*** | 0.09 | -0.12 | 0.16 |
|  | SA | 0.19 | 0.07 | 0.32*** | 0.06 | -0.27** | -0.00 |
|  | CT | 0.40*** | 0.40*** | 0.17 | 0.11 | 0.34*** | 0.16 |
| LGI | SA | 0.59*** | 0.69*** | 0.67*** | 0.68*** | 0.51*** | 0.47*** |
|  | CT | 0.20 | 0.24* | 0.06 | 0.15 | 0.03 | 0.12 |
| SA | CT | 0.05 | 0.05 | -0.03 | 0.15 | -0.19 | 0.01 |
|  | | partial correlation with age | | | | | |
| IC | LGI | 0.16 | 0.15 | 0.32*** | 0.06 | -0.23* | 0.02 |
|  | SA | 0.13 | -0.01 | 0.29*** | 0.03 | -0.33*** | -0.07 |
|  | CT | 0.24** | 0.24** | 0.03 | 0.05 | 0.25** | 0.03 |
| LGI | SA | 0.59*** | 0.68*** | 0.67*** | 0.65*** | 0.49*** | 0.49*** |
|  | CT | -0.18 | -0.12 | -0.18 | -0.07 | -0.16 | -0.04 |
| SA | CT | -0.08 | -0.11 | -0.14 | 0.01 | -0.31*** | -0.02 |
|  |  | partial correlation with sex | | | | | |
| IC | LGI | 0.26** | 0.29*** | 0.34*** | 0.08 | -0.11 | 0.16 |
|  | SA | 0.07 | 0.01 | 0.24* | 0.04 | -0.28*** | -0.08 |
|  | CT | 0.38*** | 0.40*** | 0.15 | 0.10 | 0.34*** | 0.15 |
| LGI | SA | 0.47*** | 0.58*** | 0.54*** | 0.60*** | 0.43*** | 0.28*** |
|  | CT | 0.14 | 0.22* | 0.02 | 0.08 | 0.04 | 0.07 |
| SA | CT | -0.06 | 0.00 | -0.10 | 0.06 | -0.20 | -0.06 |

Estimates from Pearsons’ (partial) correlations tests for associations between indices are shown. Top panel is results from direct correlations, panel two accounts for age and panel three accounts for sex. Those accompanied by asterisks reached statistical significance according to the adjusted significance level of *p*<0.001. **p*< 1 x 10^-3^, ***p*< 3 x 10^-4^, ****p*< 3 x 10^-5^.

**Supplementary Table 3** Results from matched analysis (n=134)

| Region | Age F | Age *p* | Sex T | Sex *p* | R^2^ (%) |
| --- | --- | --- | --- | --- | --- |
| Intrinsic Curvature | | | | | |
| Frontal | 9.43 | **2.75 x 10^-6^***** | -2.00 | 0.05 | 26 |
| Parietal | 13.57 | **1.88 x 10^-6^***** | -0.63 | 0.53 | 19 |
| Temporal | 5.37 | **4.13 x 10^-3^** | -2.29 | 0.02 | 13 |
| Occipital | 3.08 | 0.08 | -0.57 | 0.57 | 1 |
| Cingulate | 12.35 | **6.03 x 10^-4^*** | 1.90 | 0.06 | 7 |
| Insula | 25.22 | **1.55 x 10^-6^***** | -0.41 | 0.68 | 19 |
| Cortical Thickness | | | | | |
| Frontal | 55.38 | **6.19 x 10^-12^***** | -0.05 | 0.96 | 32 |
| Parietal | 60.30 | **9.02 x 10^-13^***** | -0.37 | 0.71 | 30 |
| Temporal | 36.23 | **1.40 x 10^-8^***** | -0.22 | 0.83 | 23 |
| Occipital | 52.39 | **2.01 x 10^-11^***** | 1.51 | 0.13 | 28 |
| Cingulate | 43.47 | **7.14 x 10^-10^***** | -2.19 | 0.03 | 26 |
| Insula | 24.61 | **2.04 x 10^-6^***** | 0.90 | 0.37 | 15 |
| Local Gyrification Index | | | | | |
| Frontal | 36.57 | **1.2 x 10^-8^***** | 5.17 | **8.61 x 10^-7^***** | 30 |
| Parietal | 17.79 | **1.24 x 10^-7^***** | 6.37 | **3.05 x 10^-9^***** | 35 |
| Temporal | 19.18 | **2.36 x 10^-5^***** | 6.38 | **2.89 x 10^-9^***** | 29 |
| Occipital | 8.35 | **4.51 x 10^-3^** | 5.00 | **1.86 x 10^-6^***** | 16 |
| Cingulate | 8.30 | **4.64 x 10^-3^** | 4.85 | **3.52 x 10^-6^***** | 13 |
| Insula | 7.98 | **5.47 x 10^-3^** | 6.68 | **6.41 x 10^-10^***** | 26 |
| Surface Area | | | | | |
| Frontal | 1.08 | 0.30 | 8.27 | **1.41 x 10^-13^***** | 30 |
| Parietal | 0.54 | 0.47 | 8.19 | **2.08 x 10^-13^***** | 32 |
| Temporal | 0.61 | 0.44 | 8.22 | **1.82 x 10^-13^***** | 31 |
| Occipital | 3.47 | 0.06 | 6.90 | **2.02 x 10^-10^***** | 27 |
| Cingulate | 1.08 | 0.30 | 6.57 | **1.09 x 10^-9^***** | 23 |
| Insula | 1.25 | 0.27 | 6.79 | **3.66 x 10^-10^***** | 23 |

Statistics reported are from models including sex, scanner and familiality for each index for n=134 participants who were matched across sexes (n=67 male and 67 female) by age (*t*=-0.02, *p*=0.99) and scan site (chi-squared = 0, *p*=1). R^2^ is for the full model. Adjusted significance level is *p*<0.002. **p*< 2 x 10^-3^, ***p*< 4 x 10^-4^, ****p*< 4 x 10^-5^. Similarly to analysis of the full sample inclusion of total SA in the analysis of IC, LGI and CT resulted in sex no longer having a significant effect on LGI but with otherwise comparable results.
